# A global equation-of-state model from mathematical interpolation between low- and high-density limits

**DOI:** 10.1038/s41598-022-16016-6

**Published:** 2022-07-22

**Authors:** Ti-Wei Xue, Zeng-Yuan Guo

**Affiliations:** Key Laboratory for Thermal Science and Power Engineering of Ministry of Education, Department of Engineering Mechanics, Tsinghua University, Beijing, 100084 China

**Keywords:** Engineering, Applied physics, Thermodynamics

## Abstract

The ideal gas equation of state (EOS) model is a well-known low-density limiting model. Recently, an ideal dense matter EOS model for the high-density limit symmetric to the ideal gas model has been developed. Here, by mathematically interpolating between the ideal gas and ideal dense matter limiting models, we establish a global model containing two EOS in the form of *P-V-T* and *P-S-T* for arbitrary ranges of densities. Different from empirical or semi-empirical EOS, the coefficients in the global EOS have a clear physical meaning and can be determined from a priori knowledge. The proposed global model is thermodynamically consistent and continuous. It reduces to the ideal gas model when approaching the low-density limit and to the ideal dense matter model when approaching the high-density limit. Verifications for ^4^He show that the global model reproduces the large-range behavior of matter well, along with providing important insight into the nature of the large-range behavior. Compared to the third-order virial EOS and the Benedict–Webb–Rubin EOS, the global *P-V-T* EOS has higher descriptive accuracy with fewer coefficients over a wide range of data for N_2_. The global model is shown to work well in extreme applied sciences. It predicts a linear, inverse relationship between entropy and volume when the temperature-to-pressure ratio is constant, which can explain the entropy-production behavior in shock-Hugoniots.

## Introduction

Various extreme dynamic processes such as explosive and impact loading of materials, shock wave propagation, and planetary or stellar interior evolution have attracted increasing attention. In these processes, matter may experience large ranges of densities, with correspondingly large ranges of behaviors exhibited^1^. A global equation of state (EOS) model is needed to describe these large-range behaviors of matter. It was often obtained by interpolating between different EOS models^2–7^. However, since the models used for interpolation usually cover only a restricted range of densities^1^, the models obtained by interpolating between them still cover only a restricted range of densities and are therefore not truly global. Moreover, since the models used for interpolation were usually based on different assumptions about internal structure of matter or derived by different theoretical tools, interpolating between these distinctly different models suffered from thermodynamic inconsistencies and discontinuities^1,4,6^. Consequently, the interpolation operation was more empirical^1,7^, making the interpolation EOS difficult to build or, if developed, the original physical meaning lost.

Interpolation between limits can lead to truly global results^8–11^. For example, Planck^8,9^ developed his global radiation formula available for arbitrary wavelengths based on the mathematical interpolation between the long- and short-wave limits. To obtain a truly global EOS model, the models used for interpolation should be related to the low- and high-density limits. Moreover, to satisfy thermodynamic continuity and avoid being trapped in local properties of individual substances, the models used for interpolation should be general in nature and thermodynamically compatible. As we know, the ideal gas EOS,

1 $$ PV = RT, $$

is a well-known low-density limiting model and all matter shares this simple relation at ultra-low densities^12^. Recently, it has been found that there is thermodynamic symmetry between the features of the two extremes of density, and thus an ideal dense matter EOS symmetric to the ideal gas EOS was presented^13^

2 $$ TS = R^{{\prime}} P, $$

where $R^{{\prime}}$ is a constant, called the ideal dense matter constant. It is a high-density limiting model and all matter shares this simple relation at ultra-high densities^13^. Ideal gas and ideal dense matter are a pair of symmetric theoretical concepts. Both the ideal gas and ideal dense matter models are general and they play parallel roles in thermodynamics. Here, by mathematically interpolating between them, we build a global EOS model for arbitrary ranges of densities. Two global EOS in the form of *P–V-T* and *P-S-T* are generated. The coefficients in the global EOS are all parameters related to limiting states and have a clear physical meaning. The proposed global model is thermodynamically consistent and continuous. It reduces to the original simple results in either the low- or high-density limit and thus is applicable to both low- and high-density substances. This distinctive feature of the global model enables itself to cover the large-range behavior of matter. The global model is shown to work well in extreme applied sciences such as explosive and shock-Hugoniot.

## Modeling

### Equations of state

An ideal gas, as we know, is a high-temperature and low-pressure limiting state and therefore has an infinite specific volume (low-density limit), while the ideal dense matter is a low-temperature and high-pressure limiting state and therefore has an infinitesimal specific volume (high-density limit). The actual matter shows ideal gas behavior near the low-density limit and ideal dense matter behavior near the high-density limit. The thermodynamic state of matter at intermediate densities may have some characteristics of both limiting states simultaneously and is most likely to be some kind of superposition of them. Interpolation is physically feasible.

Note that a classical interpolation technique was used for Planck’s radiation formula^8,9^. Planck saw that there were two limiting cases for radiation phenomenon, corresponding to two thermodynamic relationships: ${\text{d}}^{2} S/{\text{d}}U^{2} = {\text{a}}/U$ in the short-wave limit and ${\text{d}}^{2} S/{\text{d}}U^{2} = b/U^{2}$ in the long-wave limit. One of the critical features is that the second order derivatives of entropy with respect to internal energy have exponents of − 1 and − 2, respectively. Thus, Planck constructed the interpolation form of the two limiting relationships, ${\text{d}}^{2} S/{\text{d}}U^{2} = \upalpha /\left( {U\left( {\upbeta + U} \right)} \right)$. Combining the thermodynamic relation, ${\text{d}}S/{\text{d}}U = 1/T$, Planck eventually obtained his global radiation formula, $U =\upbeta /({\text{e}}^{{ -\upbeta /{\upalpha}T}} - 1)$.

There is a similar exponential correspondence for the low- and high-density limits of matter. The ideal gas EOS in *P-S-T* form is

3 $$ S = C_{P} \ln \frac{T}{{T_{0} }} - R\ln \frac{P}{{P_{0} }}{ + }S_{0} , $$

where $C_{P}$ is the specific heat at constant pressure and the subscript 0 denotes a given thermodynamic state. The ideal dense matter EOS in *P–V-T* form is^13^

4 $$ V = - C_{T} \ln \frac{P}{{P_{0} }} - R^{{\prime}} {\text{ln}}\frac{T}{{T_{0} }}{ + }V_{0} , $$

where $C_{T}$ is the specific work at constant temperature, defined as^14^

5 $$ C_{T} = \left( {\frac{\partial F}{{\partial P}}} \right)_{T} = - P\left( {\frac{\partial V}{{\partial P}}} \right)_{T} . $$

With pressure, *P*, and reciprocal temperature, $1/T$, as independent variables, the differentials of volume based on Eqs. (1) and (4) are

6 $$ \left\{ {\begin{array}{*{20}l} {{\text{d}}V = \left( { - RT} \right)P^{ - 2} {\text{d}}P + \left( { - \frac{R}{P}} \right)\left( \frac{1}{T} \right)^{ - 2} {\text{d}}\left( \frac{1}{T} \right)} \hfill & {\text{for ideal gas;}} \hfill \\ {{\text{d}}V = \left( { - C_{T} } \right)P^{ - 1} {\text{d}}P + \left( {R^{{\prime}} } \right)\left( \frac{1}{T} \right)^{ - 1} {\text{d}}\left( \frac{1}{T} \right)} \hfill & {{\text{for ideal dense matter}}{.}} \hfill \\ \end{array} } \right. $$

Equation (6) shows that the first order derivatives of volume with respect to pressure (as well as reciprocal temperature) have exponents of − 1 and − 2. With temperature, *T*, and reciprocal pressure, $1/P$, as independent variables, the differentials of entropy based on Eqs. (2) and (3) are

7 $$ \left\{ {\begin{array}{*{20}l} {{\text{d}}S = \left( { - R^{{\prime}} P} \right)T^{ - 2} {\text{d}}T + \left( { - \frac{{R^{{\prime}} }}{T}} \right)\left( \frac{1}{P} \right)^{ - 2} {\text{d}}\left( \frac{1}{P} \right)} \hfill & {\text{for ideal dense matter;}} \hfill \\ {{\text{d}}S = \left( {C_{P} } \right)T^{ - 1} {\text{d}}T + \left( R \right)\left( \frac{1}{P} \right)^{ - 1} {\text{d}}\left( \frac{1}{P} \right)} \hfill & {{\text{for ideal gas}}{.}} \hfill \\ \end{array} } \right. $$

Equation (7) shows that the first order derivatives of entropy with respect to temperature (as well as reciprocal pressure) also have exponents of − 1 and − 2. Interpolation between these extremes leads to a global EOS model theoretically available for arbitrary densities. For a two-degree-of-freedom thermodynamic system, two global EOS in the form of *P–V-T* and *P-S-T* are derived (See Supplementary Information)

8 $$ V = R\left( {\frac{T}{P} - \frac{{T_{0} }}{{P_{0} }}} \right) - R^{{\prime}} \ln \frac{T}{{T_{0} }} - C_{T}^{{{\text{i.s.}}}} \ln \frac{P}{{P_{0} }}{ + }V_{0} , $$

9 $$ S = R^{{\prime}} \left( {\frac{P}{T} - \frac{{P_{0} }}{{T_{0} }}} \right) - R\ln \frac{P}{{P_{0} }} + C_{P}^{{{\mathrm{i.g.}}}} \ln \frac{T}{{T_{0} }} + S_{0} , $$

where the superscript, i.s., denotes the ideal dense matter limit and the superscript, i.g., denotes the ideal gas limit. That is, the coefficients in the global EOS remain parameters with respect to the two limiting states. This is because interpolation practically operates on two limiting states and retains their original information^11^. Since the global model from interpolation removes the constraints (limiting condition) for the two limiting models, the coefficients in Eqs. (8) and (9) are superscripted to indicate that they are still parameters of the corresponding limiting states. Of course, this is not needed for the ideal gas constant, *R*, and the ideal dense matter constant, $R^{{\prime}}$, because their physical meaning is not changed in any occasion.

The global model contains the characteristics of both the ideal gas and the ideal dense matter simultaneously. Since both the ideal gas and ideal dense matter models are general, it has generality. The proposed global model is thermodynamically consistent and continuous. It reduces to the ideal gas model when approaching the low-density (high-temperature and low-pressure) limit and to the ideal dense matter model when approaching the high-density (high-pressure and low-temperature) limit, which suggests that the global model can describe the physical properties of either the low- or high-density matter. This distinctive feature of the global model enables itself to cover the large-range behavior of matter experiencing a large range of densities. Yet, since both limiting models can only be applied to homogeneous substances not located in a region with rapid property variations such as near the critical point or the two-phase region, then the global model is not expected to apply in those regions either. The global model reflects the commonality of thermodynamic behavior of matter and could serve as a basis to determine the physical properties of individual substances.

### Characteristics

With temperature and pressure as independent variables, the differentials of volume and entropy are derived based on Eqs. (8) and (9), respectively

10 $$ \left\{ \begin{gathered} {\text{d}}V = \left( { - \frac{RT}{{P^{2} }} - \frac{{C_{T}^{{{\text{i.s.}}}} }}{P}} \right){\text{d}}P + \left( {\frac{R}{P} - \frac{{R^{{\prime}} }}{T}} \right){\text{d}}T; \hfill \\ {\text{d}}S = \left( {\frac{{R^{{\prime}} }}{T} - \frac{R}{P}} \right){\text{d}}P + \left( { - \frac{{R^{{\prime}} P}}{{T^{2} }} + \frac{{C_{P}^{{{\mathrm{i.g.}}}} }}{T}} \right){\text{d}}T. \hfill \\ \end{gathered} \right. $$

Equation (10) shows a reciprocal relation between Eqs. (8) and (9), i.e., the Maxwell’s relation corresponding to the Gibbs free energy,

11 $$ \left( {\frac{\partial V}{{\partial T}}} \right)_{P} = - \left( {\frac{\partial S}{{\partial P}}} \right)_{T} = \frac{R}{P} - \frac{{R^{{\prime}} }}{T}. $$

This reflects that Eqs. (8) and (9) are is thermodynamically compatible, and also indicates to some extent the thermodynamic compatibility between the ideal gas and ideal dense matter models. Together Eqs. (8) and (9) provide a complete description for a thermodynamic system. The expressions for various thermodynamic variables can be derived based on them.

Derive the differential expression for internal energy based on Eq. (10)

12 $$ \begin{aligned} {\text{d}}U & = \left( {C_{P}^{{{\mathrm{i.g.}}}} - R} \right){\text{d}}T + \left( {C_{T}^{{{\text{i.s.}}}} + R^{{\prime}} } \right){\text{d}}P \\ & = C_{V}^{{{\mathrm{i.g.}}}} {\text{d}}T + C_{S}^{{{\text{i.s.}}}} {\mathrm{d}}P, \\ \end{aligned} $$

where $C_{S}$ is the specific work at constant entropy, defined as^14^

13 $$ \, C_{S} = \left( {\frac{\partial U}{{\partial P}}} \right)_{S} = - P\left( {\frac{\partial V}{{\partial P}}} \right)_{S} . $$

There is a parametric relationship between $C_{S}$ and $C_{T}$ for ideal dense matter^13^

14 $$ C_{T} = C_{S} - R^{{\prime}} . $$

Equation (12) reveals how these two limiting parameters, $C_{V}^{{{\mathrm{i.g.}}}}$ and $C_{S}^{{{\text{i.s.}}}}$, exist in the physical properties of actual matter

15 $$ C_{V}^{{{\mathrm{i.g.}}}} = \left( {\frac{\partial U}{{\partial T}}} \right)_{P} {;}\quad C_{S}^{{{\text{i.s.}}}} = \left( {\frac{\partial U}{{\partial P}}} \right)_{T} . $$

That is, the specific heat at constant volume in the ideal gas limit is exactly the partial derivative of internal energy of actual matter with respect to temperature under isobaric conditions and the specific work at constant entropy in the ideal dense matter limit is exactly the partial derivative of internal energy of actual matter with respect to pressure under isothermal conditions. Further derive the expressions for specific heats as well as specific works

16 $$ \left\{ {\begin{array}{*{20}l} {C_{V} = T\left( {\frac{\partial S}{{\partial T}}} \right)_{V} = C_{V}^{{{\mathrm{i.g.}}}} + C_{S}^{{{\text{i.s.}}}} \frac{{R - R^{{\prime}} \frac{P}{T}}}{{C_{T}^{{{\text{i.s.}}}} + R\frac{T}{P}}};} \hfill \\ {C_{P}  = T\left( {\frac{\partial S}{{\partial T}}} \right)_{P} = C_{P}^{{{\mathrm{i.g.}}}} - R^{{\prime}} \frac{P}{T};} \hfill \\ {C_{S}  =  - P\left( {\frac{\partial V}{{\partial P}}} \right)_{S} = C_{S}^{{{\text{i.s.}}}} - C_{V}^{{{\mathrm{i.g.}}}} \frac{{R^{{\prime}} - R\frac{T}{P}}}{{C_{P}^{{{\mathrm{i.g.}}}} - R^{{\prime}} \frac{P}{T}}};} \hfill \\ {C_{T}  =  - P\left( {\frac{\partial V}{{\partial P}}} \right)_{T} = C_{T}^{{{\text{i.s.}}}} + R\frac{T}{P}.} \hfill \\ \end{array} } \right. $$

Equation (16) shows the general characteristics of specific heats and specific works that are also the relationships with their limiting counterparts. According to Eq. (16), the specific heats as well as the specific works are all a function of the temperature-to-pressure ratio, $T/P$, (or the pressure-to-temperature ratio, $P/T$) only. The specific heats reduce to the corresponding ones of ideal gas in the high-temperature and low-pressure limit, $T/P \to \infty$, and the specific works reduce to the corresponding ones of ideal dense matter in the high-pressure and low-temperature limit, $T/P \to 0$.

The limiting parameters in the global EOS, in turn, can be determined by other accessible parameters. For example, except for the known parameter, *R*, the other three limiting parameters can be derived inversely based on the expressions for the specific heats and specific works

17 $$ \left\{ {\begin{array}{*{20}l} {C_{P}^{{{\mathrm{i.g.}}}} = C_{P} + R - \sqrt {C_{T} (C_{P} - C_{V} )\frac{P}{T}} ;} \hfill \\ {C_{T}^{{{\text{i.s.}}}} = C_{T} - R\frac{T}{P};} \hfill \\ {R^{{\prime}} = R\frac{T}{P} - \sqrt {C_{P} (C_{T} - C_{S} )\frac{T}{P}} .} \hfill \\ \end{array} } \right. $$

$C_{V}$ and $C_{P}$ are common property parameters for which there are abundant experimental data. The values of $C_{S}$ and $C_{T}$ are accessed from the isentropic compressibility coefficient, $\kappa_{S}$, and the isothermal compressibility coefficient, $\kappa_{T}$, respectively

18 $$ C_{S} = PV\kappa_{S} {;}\quad C_{T} = PV\kappa_{T} . $$

Therefore, the coefficients in the global EOS are available a priori. In this sense, the global model is an a priori model.

## Verifications

### Verifying specific heats and specific works

**Figure 1:**
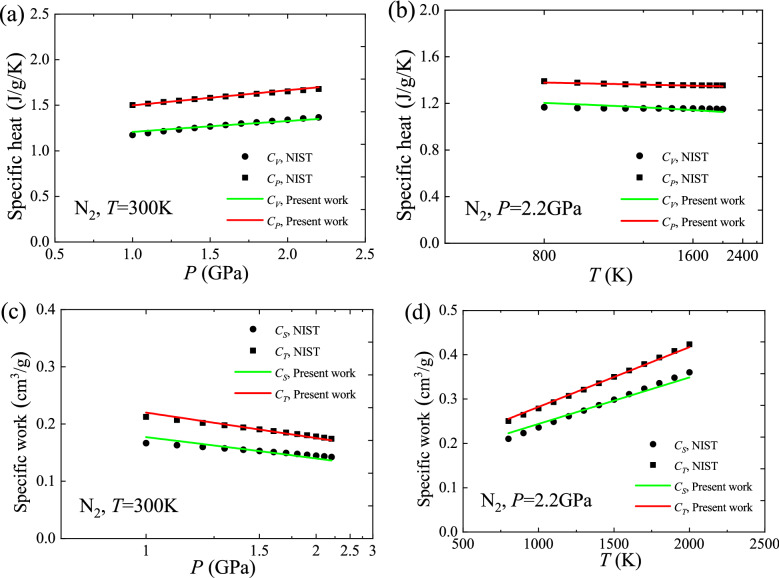
Verification of the specific heats and specific works using N_2_ data. (**a**) Specific heats versus pressure at 300 K; (**b**) Specific heats versus reciprocal temperature at 2.2 GPa; (**c**) Specific works versus reciprocal pressure at 300 K; (**d**) Specific works versus temperature at 2.2 GPa.

The specific heats and specific works of N_2_ are calculated using Eq. (16) without free-fitting parameters. Figure 1 shows that these calculated values match well the values from the REFPROP program (DLL version 9.1) developed by the National Institute of Standards and Technology (NIST). The relative deviations are less than 1.1% for $C_{P}$, less than 3.2% for $C_{V}$, less than 3.5% for $C_{T}$, and less than 6.4% for $C_{S}$. In addition, the global model predicts that under isothermal conditions, $C_{P}$ is linear with respect to pressure and $C_{T}$ is linear with respect to reciprocal pressure; under isobaric conditions, $C_{P}$ is linear with respect to reciprocal temperature and $C_{T}$ is linear with respect to temperature, all of which are consistent with the actual features exhibited for N_2_. This supports the correctness of the global model.

### Verifying two global EOS

**Figure 2:**
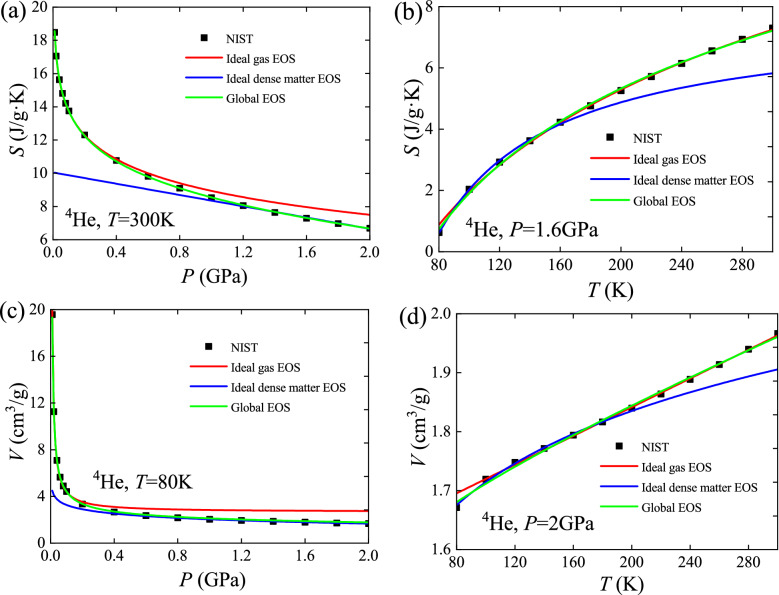
Verification of the global EOS using ^4^He data and comparison with the ideal gas and ideal dense matter EOS. (**a**) Entropy versus pressure at 300 K; (**b**) Entropy versus temperature at 1.6 GPa; (**c**) Volume versus pressure at 80 K; (**d**) Volume versus temperature at 2 GPa.

These two global EOS, Eqs. (8) and (9), are verified using ^4^He data and compared with the ideal gas and ideal dense matter EOS (Fig. 2). The zero-value point of entropy for selected data is the normal boiling point (NBP). The coefficients in the two global EOS are calculated by Eq. (17). The ideal gas EOS are extrapolated to the high-pressure or low-temperature region from correlations of data in the low-pressure or high-temperature region. Results show that both the entropy and volume predicted by the ideal gas EOS are always larger than the data from NIST. The ideal dense matter EOS are extrapolated to the low-pressure or high-temperature region from correlations of data in the high-pressure or low-temperature region. Results show that both the entropy and volume predicted by the ideal dense matter EOS are always smaller than the data from NIST. The ideal gas and ideal dense matter EOS give upper and lower limits for thermodynamic behavior of matter, respectively. Compared with them, the global EOS reproduce the entire selected property data excellently, which indicates that the large-range behavior of matter at intermediate densities can be explained by a superposition of the properties of the low- and high-density limits. The interpolation pattern correctly reveals the respective proportions of the contribution from these two limiting states.

## Applications

### Interpolation versus extrapolation

Extrapolation based on the ideal gas limit has been a common approach to developing an EOS for actual matter^15,16^. However, the EOS yielded by this approach were mostly empirical or semi-empirical for high-density matter due to the complexity and diversity of their molecular interactions^17–22^. These EOS usually have complex forms or plenty of empirical coefficients. These coefficients are “isolated” and their values can only be determined from a large amount of experimental data. When these EOS are further extrapolated beyond the range where they were fitted to experimental data, the results are generally not reliable^23–25^.

**Figure 3:**
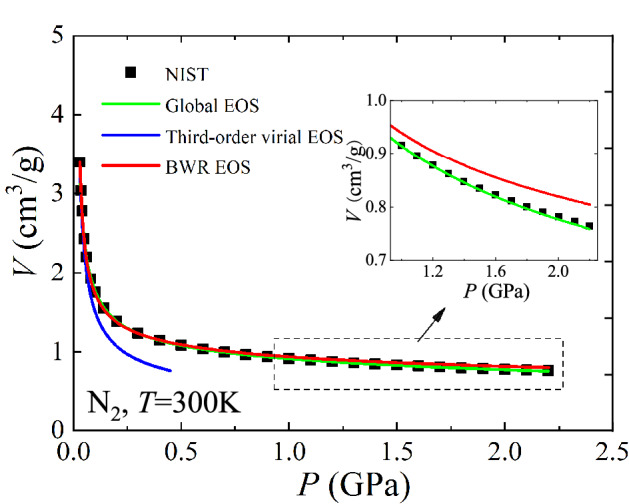
Comparison of the global *P-V-T* EOS with the third-order virial EOS and the BWR EOS using N_2_ data.

Extrapolation only yields a local result, while interpolation can lead to a global result. In the following, the global *P-V-T* EOS, Eq. (8), from interpolation is compared with the third-order virial EOS and the Benedict-Webb-Rubin (BWR) EOS from extrapolation using N_2_ data at 300 K (Fig. 3). The values of coefficients in the third-order virial EOS and the BWR EOS are taken from the work of Nowak et al.^26^ and Crain Jr et al.^27^, respectively. Results show that the third-order virial EOS with three coefficients is valid only for relatively low-pressure region. The BWR EOS was extrapolated to relatively high-pressure region by introducing eight empirical coefficients. Even so, the accuracy of the BWR EOS decreases as the pressure increases further. Since both the third-order virial EOS and the BWR EOS were constructed with the ideal gas EOS as the basic framework, their extrapolation behaviors are certainly deteriorating with increasing pressure or density. Compared to the third-order virial EOS and the BWR EOS, the global *P-V-T* EOS shows a significant advantage by using both the ideal gas and ideal dense matter EOS as the basic framework. It achieves higher descriptive accuracy with fewer coefficients (two coefficients for isothermal conditions) over a wide range of data for N_2_, especially in the ultrahigh-pressure region. Additionally, the empirical coefficients in the virial EOS and the BWR EOS need to be determined by fitting a large amount of experimental data^26,27^, while the coefficients in the global *P-V-T* EOS have a clear thermodynamic meaning and can be determined from a priori knowledge.

### Explosive physics

**Figure 4:**
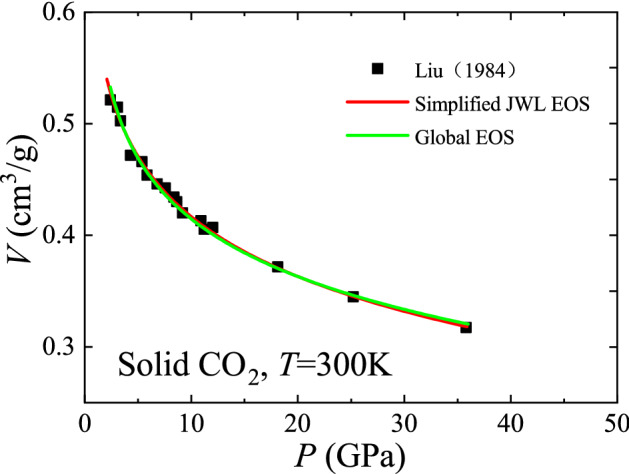
Comparison of the global *P-V-T* EOS with the simplified JWL EOS using solid CO_2_ data.

The Jones–Wilkins–Lee (JWL) EOS is a well-known empirical EOS in explosive physics and is quite effective in describing the expansion behavior of the detonation products of condensed explosives^28,29^. It is pressure-explicit and has a simplified form,

19 $$ P = A{\text{e}}^{{ - R_{1} V}} + \frac{RT}{V}, $$

where *A* and *R*_1_ are constants. The first term on the right side of Eq. (19) represents the high-pressure contribution and the second term represents the low-pressure contribution. The former is actually the isothermal form of the ideal dense matter *P-V-T* EOS, while the latter expresses the ideal gas EOS. Therefore, the simplified JWL EOS can be understood as a superposition of the ideal dense matter state and the ideal gas state under the pressure representation. As a comparison, the global *P-V-T* EOS is volume-explicit and behaves as a superposition of the ideal dense matter state and the ideal gas state under the volume representation. Figure 4 shows further comparison between the global *P-V-T* EOS and the simplified JWL EOS using the solid CO_2_ data from the high-pressure experiment of Liu^30^. Results show that both are in good agreement with the isothermal data of solid CO_2_. However, with respect to the effect of temperature, the simplified JWL EOS retains only the ideal gas part, while the global *P-V-T* EOS keeps both the ideal dense matter part and the ideal gas part. Therefore, the global *P-V-T* EOS may reflect the effect of temperature more accurately. In addition, as mentioned earlier, the coefficients in the global *P-V-T* EOS can be obtained a priori, while the simplified JWL EOS has empirical coefficients that need to be experimentally fitted.

### Shock-Hugoniots

**Figure 5:**
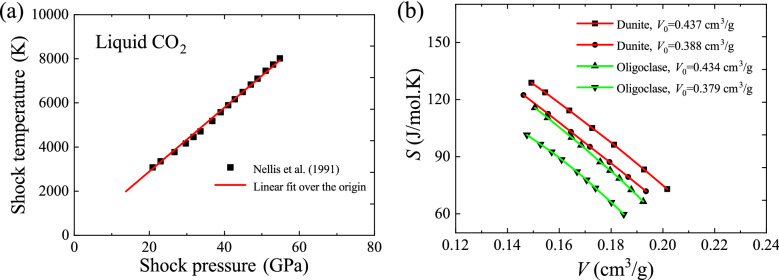
Shock-Hugoniots. (**a**) Shock temperature versus shock pressure for liquid CO_2_; (**b**) entropy versus volume for dunite and oligoclase, where *V*_0_ is initial volume.

Combining Eqs. (8) and (9) yields a new global EOS,

20 $$ C_{S}^{{{\text{i.s.}}}} S + C_{V}^{{{\mathrm{i.g.}}}} V = C_{S}^{{{\text{i.s.}}}} R^{{\prime}} \frac{P}{T} + C_{V}^{{{\mathrm{i.g.}}}} R\frac{T}{P} + \left( {C_{S}^{{{\text{i.s.}}}} C_{V}^{{{\mathrm{i.g.}}}} + C_{S}^{{{\text{i.s.}}}} R - C_{V}^{{{\mathrm{i.g.}}}} R^{{\prime}} } \right)\ln \frac{T}{P} + {\text{c}}, $$

where c is an integration constant. Equation (20) has a particular feature that temperature and pressure are in the form of a ratio, $T/P$ (or $P/T$). When the temperature-to-pressure ratio is kept constant, a very simple linear relationship between entropy and volume appears

21 $$ S = - \frac{{C_{V}^{{{\mathrm{i.g.}}}} }}{{C_{S}^{{{\mathrm{i.s.}}}}}}V + {\text{C}}, $$

where C is a constant. This new physical property predicted by the global model can be used to describe the shock adiabatic compression (Hugoniot). The temperature-to-pressure ratio is approximately constant for a large number of shock adiabatic processes^31–34^. For example, the shock experiments of Nellis et al.^35^ on liquid CO_2_ shows that the shock temperature is almost linearly proportional to the shock pressure (Fig. 5a). Therefore, according to Eq. (21), the entropy of shock adiabatic process should have linear, inverse relationship with volume, which is consistent with the observations of Ahrens et al.^36^ for minerals such as dunite and oligoclase (Fig. 5b). This suggests that the shock-induced entropy production depends directly on the degree of volume compression.

## Conclusions

Interpolation between the low- and high-density limits is needed to gain a truly global EOS model. The ideal gas model is a well-known model for the low-density limit. Recently, an ideal dense matter model for the high-density limit symmetric to the ideal gas model has been developed. In this work, by mathematically interpolating between the ideal gas and ideal dense matter limiting models, we establish a global model containing two EOS in the form of *P-V-T* and *P-S-T* for arbitrary ranges of densities. The two global EOS constitute a complete description for the thermodynamic properties of matter.

The proposed global model is thermodynamically consistent and continuous. It returns to the ideal gas model when approaching the low-density limit and to the ideal dense matter model when approaching the high-density limit. The global model can be applied to either low- or high-density matter, and thus can cover the large-range behavior of matter. Verification for ^4^He shows that the ideal gas model always gives larger predicted values of entropy and volume, and the ideal dense matter model always gives smaller predicted values, while the global model reproduces well the property data of ^4^He experiencing a large range of densities. The thermodynamic behaviors of matter at intermediate densities show the physical nature of superposition of the low- and high-density limits.

Since interpolation uses information only on the low- and high-density limits, the coefficients in the global EOS are all parameters of limiting states with a clear physical meaning. They can be determined from a priori knowledge. In this sense, the global model is an a priori model. The general expressions for specific heats and specific works are derived and their values for N_2_ are calculated without free-fitting parameters. The calculated values are verified to be in good agreement with the values from the NIST database.

The global model is shown to work well in applied sciences dealing with high-density matter. Compared to the third-order virial EOS and the BWR EOS, the global *P-V-T* EOS achieves higher descriptive accuracy with fewer coefficients over a wide range of data for N_2_, especially in the ultrahigh-pressure region. Interpolation between the ideal gas and ideal dense matter models shows significant advantages over extrapolation on the ideal gas model only. The global *P-V-T* EOS is also shown to have almost the same effect as the classical JWL EOS in describing the expansion behavior of detonation products. Furthermore, the global model predicts a linear, inverse relationship between entropy and volume when the temperature-to-pressure ratio is constant, which can explain the entropy-production behavior in shock- Hugoniots.

## Supplementary Information

Supplementary Information.

## Data Availability

The data that support the findings of this study are available from the corresponding author upon reasonable request.
