# Supplementary material for: A global equation-of-state model from mathematical interpolation between low- and high-density limits
Source: Sci Rep. 2022 Jul 22;12:12533. doi: 10.1038/s41598-022-16016-6 (PMC9307579; doi:10.1038/s41598-022-16016-6)
Supplement: Supplementary file 1 — Supplementary Information. [file 41598_2022_16016_MOESM1_ESM.pdf]

A global equation-of-state model from mathematical interpolation between low- and high-density limits

Ti-Wei Xue and Zeng-Yuan Guo*

Key Laboratory for Thermal Science and Power Engineering of Ministry of Education, Department of Engineering Mechanics, Tsinghua University, Beijing 100084, China

* Correspondence: demgzy@tsinghua.edu.cn

Derivation of global EOS

Note that interpolation satisfying physical rationality has a unique form, which will be shown in the derivation below. Based on Eq. (6), the first order derivatives of pressure with respect to volume at constant temperature are

$$\begin{cases} \left(\frac{\partial P}{\partial V}\right)_T = -\frac{1}{C_T}\frac{1}{\left(\frac{1}{P}\right)} & \text{for ideal dense matter;} \\ \left(\frac{\partial P}{\partial V}\right)_T = -\frac{1}{RT}\frac{1}{\left(\frac{1}{P}\right)^2} & \text{for ideal gas.} \end{cases} \tag{S1}$$

The interpolation between these two extremes gives

$$\left(\frac{\partial P}{\partial V}\right)_T = -\frac{1}{C_T^{\text{i.s.}}}\left(\frac{1}{\frac{1}{P}} - \frac{1}{\frac{1}{P}+\frac{C_T^{\text{i.s.}}}{RT}}\right) = -\frac{1}{RT}\frac{1}{\frac{1}{P}\left(\frac{1}{P}+\frac{C_T^{\text{i.s.}}}{RT}\right)}, \tag{S2}$$

where the superscript i.s. denotes ideal dense matter. Under isothermal conditions, Eq. (S2) reduces to the ideal gas part of Eq. (S1) when the pressure tends to zero and to the ideal dense matter part when the pressure tends to infinity, which confirms the physical correctness of the above interpolation operation. The integral of Eq. (S2) with respect to pressure yields

$$V = \frac{RT}{P} - \frac{RT}{P_0} - C_T^{\text{i.s.}} \ln\frac{P}{P_0} + V\left(T, P_0\right), \tag{S3}$$

where $V\left(T, P_0\right)$ is an unknown function of temperature. Therefore, another interpolation is needed to relate temperature and volume. The first order derivatives of $1/T$ with respect to volume at constant pressure based on Eq. (6) are

$$
\begin{cases}
\left[\dfrac{\partial\left(\dfrac{1}{T}\right)}{\partial V}\right]_P = \dfrac{1}{R'}\dfrac{1}{T} & \text{for ideal dense matter;} \\
\left[\dfrac{\partial\left(\dfrac{1}{T}\right)}{\partial V}\right]_P = -\dfrac{P}{R}\dfrac{1}{T^2} & \text{for ideal gas.}
\end{cases}
\tag{S4}
$$

Then interpolation gives

$$
\left[\frac{\partial\left(\frac{1}{T}\right)}{\partial V}\right]_P = \frac{1}{R'}\left(\frac{1}{T}-\frac{1}{T-R'\frac{P}{R}}\right) = -\frac{P}{R}\frac{1}{T\left(T-R'\frac{P}{R}\right)}. \tag{S5}
$$

Under isobaric conditions, Eq. (S5) reduces to the ideal gas part of Eq. (S4) when the temperature tends to infinity and to the ideal dense matter part when the temperature tends to zero. The integral of Eq. (S5) with respect to temperature yields

$$
V = \frac{RT}{P} - \frac{RT_0}{P} - R'\ln\frac{T}{T_0} + V\left(T_0, P\right). \tag{S6}
$$

Combining Eqs. (S3) and (S6) yields

$$
-\frac{RT}{P_0} - C_T^{\text{i.s.}}\ln\frac{P}{P_0} + V\left(T, P_0\right) = -\frac{RT_0}{P} - R'\ln\frac{T}{T_0} + V\left(T_0, P\right) \tag{S7}
$$

Moving the terms in Eq. (S7) yields

$$
V\left(T, P_0\right) - \frac{RT}{P_0} + R'\ln\frac{T}{T_0} = V\left(T_0, P\right) - \frac{RT_0}{P} + C_T^{\text{i.s.}}\ln\frac{P}{P_0}. \tag{S8}
$$

The left side of the equal sign in Eq. (S8) is simply a function of temperature, while the right side is simply a function of pressure. Therefore, they can only be equal to the same constant. Then, the two unknown functions, $V\left(T, P_0\right)$ and $V\left(T_0, P\right)$, are determined

$$
\begin{cases}
V\left(T, P_0\right) = \dfrac{RT}{P_0} - R'\ln\dfrac{T}{T_0} + \mathrm{c}; \\
V\left(T_0, P\right) = \dfrac{RT_0}{P} - C_T^{\text{i.s.}}\ln\dfrac{P}{P_0} + \mathrm{c};
\end{cases}
\tag{S9}
$$

where c is a constant. Substituting the expression for $V\left(T, P_0\right)$ in Eq. (S9) into Eq. (S3) yields

$$V=\frac{RT}{P}-R'\ln\frac{T}{T_0}-C_T^{\text{i.s.}}\ln\frac{P}{P_0}+\text{c}. \tag{S10}$$

Of course, substituting the expression of $V\left(T_0,P\right)$ in Eq. (S9) into (S6) can also yield Eq. (S10). When $V=V_0$, Eq. (S10) becomes

$$V_0=\frac{RT_0}{P_0}+\text{c}. \tag{S11}$$

Substituting Eq. (S11) into (S10) yields the final global EOS in *P*-*V*-*T* form,

$$V=R\left(\frac{T}{P}-\frac{T_0}{P_0}\right)-R'\ln\frac{T}{T_0}-C_T^{\text{i.s.}}\ln\frac{P}{P_0}+V_0. \tag{S12}$$

The same approach is used to derive the global EOS in *P*-*S*-*T* form. Based on Eq. (7), the first order derivatives of temperature with respect to entropy at constant pressure are

$$\begin{cases}\left(\frac{\partial T}{\partial S}\right)_P=-\frac{1}{R'P}\frac{1}{\left(\frac{1}{T}\right)^2} & \text{for ideal dense matter;}\\ \left(\frac{\partial T}{\partial S}\right)_P=\frac{1}{C_P}\frac{1}{\left(\frac{1}{T}\right)} & \text{for ideal gas.}\end{cases} \tag{S13}$$

Then, the interpolation of these two equations gives

$$\left(\frac{\partial T}{\partial S}\right)_P=\frac{1}{C_P^{\text{i.g.}}}\left(\frac{1}{\frac{1}{T}}-\frac{1}{\frac{1}{T}-\frac{C_P^{\text{i.g.}}}{R'P}}\right)=-\frac{1}{R'P}\frac{1}{\frac{1}{T}\left(\frac{1}{T}-\frac{C_P^{\text{i.g.}}}{R'P}\right)}, \tag{S14}$$

where the superscript i.g. denotes ideal gas. The integral of Eq. (S14) with respect to temperature yields

$$S=\frac{R'P}{T}-\frac{R'P}{T_0}+C_P^{\text{i.g.}}\ln\frac{T}{T_0}+S\left(T_0,P\right). \tag{S15}$$

The first order derivatives of $1/P$ with respect to entropy at constant temperature based on Eq. (7) are

$$
\begin{cases}
\left[\dfrac{\partial\left(\dfrac{1}{P}\right)}{\partial S}\right]_T = -\dfrac{T}{R'}\dfrac{1}{P^2} & \text{for ideal dense matter;} \\
\left[\dfrac{\partial\left(\dfrac{1}{P}\right)}{\partial S}\right]_T = \dfrac{1}{R}\dfrac{1}{P} & \text{for ideal gas.}
\end{cases} \tag{S16}
$$

The interpolation between these two equations gives

$$
\left[\frac{\partial\left(\dfrac{1}{P}\right)}{\partial S}\right]_T = \frac{1}{R}\left(\frac{1}{P}-\frac{1}{P-R\dfrac{T}{R'}}\right) = -\frac{T}{R'}\frac{1}{P\left(P-R\dfrac{T}{R'}\right)}. \tag{S17}
$$

The integral of Eq. (S17) with respect to pressure yields

$$
S = \frac{R'P}{T} - \frac{R'P_0}{T} - R\ln\frac{P}{P_0} + S\left(T, P_0\right). \tag{S18}
$$

Combining Eqs. (S15) and (S18) with the same mathematical technique as before yields the final global EOS in *P*-*S*-*T* form,

$$
S = R'\left(\frac{P}{T}-\frac{P_0}{T_0}\right) - R\ln\frac{P}{P_0} + C_P^{\text{i.g.}}\ln\frac{T}{T_0} + S_0. \tag{S19}
$$
